# Association between sarcopenia and functionality in older adults in the Peruvian Amazon

**DOI:** 10.17843/rpmesp.2026.433.16113

**Published:** 2026-09-29

**Authors:** Fabrizio Carlos-Mallorga, Jarumi M. Flores-Samamé, Naskia L. Valdivia-Salas, Alvaro M. Ñaña-Cordova, Xiomara C. Benavente-Chalco, Kiara Camacho-Caballero, José F. Parodi, Fernando M. Runzer-Colmenares

**Affiliations:** 1 Carrera de Medicina Humana, Universidad Científica del Sur. Lima, Peru. Universidad Científica del Sur Carrera de Medicina Humana Universidad Científica del Sur Lima Peru; 2 CHANGE Research Working Group, Facultad de Ciencias de la Salud, Universidad Científica del Sur. Lima, Peru. Universidad Científica del Sur CHANGE Research Working Group Facultad de Ciencias de la Salud Universidad Científica del Sur Lima Peru; 3 Centro de Investigación del Envejecimiento (CIEN), Facultad de Medicina Humana, Universidad de San Martín de Porres. Lima, Peru. Universidad de San Martín de Porres Centro de Investigación del Envejecimiento (CIEN) Facultad de Medicina Humana Universidad de San Martín de Porres Lima Peru

**Keywords:** Sarcopenia, Functional Dependence, Muscle Strength, Aging, Amazon

## Abstract

**Objectives.:**

To analyze the relationship between sarcopenia and functional dependence in older adults in the Peruvian Amazon.

**Materials and methods.:**

A cross-sectional study was conducted based on a secondary data analysis of the Amazon Frail study that included older adults (60 years or older). Sarcopenia was assessed using the criteria established by the Asian Working Group for Sarcopenia, which include the measurement of muscle strength, muscle mass, and physical performance; and functionality, using the Barthel scale. In addition, sociodemographic and anthropometric characteristics, as well as medical and personal history, were included. A Poisson regression analysis with robust variance was performed to estimate crude and adjusted prevalence ratios (PR), with their respective 95% confidence intervals (95%CI).

**Results.:**

A total of 315 older adults were included in the analysis, of whom 193 (n=61.3%) were women. It was found that 113 (n=35.9%) presented sarcopenia and 115 (n=36.5%) had functional dependence. In the adjusted Poisson regression analysis, it was found that sarcopenia was significantly associated with a higher probability of functional dependence (PR=1.43; 95%CI: 1.05-1.95). Furthermore, frailty (PR=2.07; 95%CI: 1.27-3.38), subjective memory complaint (PR=1.40; 95%CI: 1.02-1.91), and hearing loss (PR=1.53; 95%CI: 1.09-2.14) were also factors associated with greater functional dependence.

**Conclusions.:**

In rural Amazonian communities, sarcopenia in older adults is associated with functional dependence. Its early identification is fundamental to prevent functional dependence and guide comprehensive health interventions.

## INTRODUCTION

The sustained increase in life expectancy has oriented attention toward the concept of healthy aging, understood as the preservation of functionality in older adults ^1^. Functionality is defined as the ability to live independently, perform basic activities, and assume self-care, with dependence in basic activities of daily living (BADL) being one of the most severe manifestations of functional deterioration in older adults. ^1^. Internationally, prevalences of moderate and severe dependence of 10.2% and 4.8% toward activities of daily living, respectively, have been reported ^2^. However, the alteration of functionality is not homogeneous and varies according to the comorbidity burden and care context. For example, in high-Andean rural areas of Peru, a functional dependence prevalence of 51.1% in basic activities of daily living in older adults has been documented, a figure that anticipates a high demand for healthcare resources in the medium term ^3^. This finding acquires additional relevance if it is considered that functional dependence has been associated with higher hospital mortality in this population ^4^. Although functional dependence is usually a gradual and progressive process, the appearance of medical conditions leading to dependence in older adults continues to represent a priority public health challenge.

Among the conditions closely linked to aging is sarcopenia, a multifactorial geriatric syndrome characterized by the progressive and generalized loss of muscle mass, accompanied by a decrease in strength and physical performance ^1^^,^^5^. This condition increases vulnerability and the risk of dependence in older adults when not timely identified and intervened ^1^. Its prevalence markedly increases with age, ranging between 5% and 13% in adults aged 60 to 70 years and exceeding 50% in those over 80 years ^5^. In the context of increasing life expectancy, it is predictable that the burden of sarcopenia will also rise, given that muscle strength begins to decline from the third decade of life. This complicates its clinical approach and constitutes a relevant challenge due to its direct impact on the functionality and autonomy of older adults ^6^.

Given that sarcopenia can accelerate the onset of functional dependence, it is fundamental to evaluate its possible associations considering the demographic, cultural, and clinical particularities of each population. In Peru, a hospital study reported a sarcopenia prevalence of 49.4%, which evidences the magnitude of the problem in the clinical setting ^7^. Likewise, it has been described that the presence of sarcopenia increases by 1.58 times the risk of developing functional dependence ^8^. However, the available evidence on sarcopenia and functional decline in older adults residing in rural areas continues to be limited. Most national research has focused on Andean rural populations, in which a sarcopenia prevalence of 17.6% and a significant association between this condition and functional dependence has been documented ^9^.

The geriatric population of the Peruvian Amazon, despite presenting particular environmental, sociocultural, and nutritional characteristics, remains scarcely explored in the scientific literature. In this context, the objective of the present study was to analyze the relationship between sarcopenia and functional dependence in older adults residing in the Peruvian Amazon.

**KEY MESSAGES**

Motivation for the study. In the Peruvian Amazon, many older adults live in rural communities, with difficult displacements, and limited access to health services. In this context, little is known about how sarcopenia affects their daily lives.

Main findings. We evaluated older adults living in rural Amazonian communities and found that sarcopenia was frequent. Those who presented it were more likely to need help with bathing, dressing, or mobilizing.

Public health implications. It is fundamental to detect sarcopenia early. Community exercise, nutrition, and family support programs, adapted to the Amazonian geographical and cultural reality, are required to protect the autonomy and quality of life of older adults.

## MATERIALS AND METHODS

### Design and context of the study

An observational, analytical, cross-sectional, and retrospective study was conducted, corresponding to a secondary analysis of data from the Amazon Frail ^10^ study. Data were collected between March and July 2024 in rural communities of the Peruvian Amazon. The original study aimed to evaluate the state of frailty and functional dependence in older adults residing in these communities. Participants were selected through a non-probabilistic census-type sampling and included individuals aged 60 years or older residing in the evaluated communities ^10^. This manuscript was prepared following the recommendations of the Strengthening the Reporting of Observational Studies in Epidemiology (STROBE) guideline for cross-sectional observational studies.

### Participants

For the original study, individuals aged 60 years or older were included and participants who presented moderate or severe cognitive impairment, defined by eight or more errors on the Pfeiffer questionnaire, visual or auditory deficit limiting the performance of physical evaluations or the interview, refusal to participate, or difficulties expressing themselves or understanding the Spanish language ^10^ were excluded.

For the present secondary analysis, only records that had complete information on the variables of interest sarcopenia and functionality were included. Records with incomplete data in any of the main variables were excluded. No missing data were identified in the variables of interest, so all eligible records were included in the final analysis, reaching a sample of 315 participants.

### Main Variables

The outcome variable was dependence in basic activities of daily living (BADL), evaluated using the Barthel index. This instrument assesses functional capacity to perform 10 basic activities of daily living, with scores ranging between 0 and 100 points ^11^. Participants with scores ≤80 were classified as functionally dependent, while those with scores >80 were considered functionally independent ^11^.

The exposure variable was sarcopenia, evaluated following the diagnostic criteria proposed by the Asian Working Group for Sarcopenia 2019 (AWGS) ^(^^12^, which consider the evaluation of muscle strength, muscle mass, and physical performance.

Muscle strength was evaluated by measuring the handgrip strength of the dominant hand using a Jamar® hydraulic dynamometer. Decreased muscle strength was considered a value ≤28 kg in men and ≤18 kg in women, according to the cutoff points established by the AWGS ^12^.

Muscle mass was estimated by electrical bioimpedance analysis (BIA) using the BodyStat®500 device (Bodystat LTD, Douglas, Isle of Man, UK), single-frequency (50 kHz), with the participant in a supine position. Appendicular skeletal muscle mass (ASMM) was estimated using the following equation: ASMM (kg) = -0.05376 + (0.2394 × H²/R) + (2.708 × sex) + (0.065 × weight) where H corresponds to height (cm), R to the resistance measured at 50 kHz and sex was coded as 1 for men and 0 for women. Subsequently, the skeletal muscle mass index (SMI = ASMM/height²) was calculated, with low muscle mass considered for values ≤7.0 kg/m² in men and ≤5.7 kg/m² in women ^13^.

Physical performance was evaluated using the Short Physical Performance Battery (Short Physical Performance Battery, SPPB), which includes balance tests, 4-meter gait speed, and standing up from a chair. Low physical performance was considered a score ≤9 points ^12^.

Sarcopenia was classified as the simultaneous presence of decreased muscle strength and muscle mass (confirmed sarcopenia), as well as the concomitant presence of decreased muscle strength, muscle mass, and physical performance (severe sarcopenia). For the analysis, both categories were grouped into a single dichotomous variable (presence/absence of sarcopenia).

### Covariables

The identification of the set of covariates was performed using a directed acyclic graph (DAG) (supplementary material).

Sociodemographic variables were included such as sex (male/female), age (years), marital status (single, married/cohabiting, divorced/separated/widowed), educational level (illiterate/incomplete primary/complete primary/incomplete secondary/complete secondary/technical education/higher education) and social support, evaluated using a question from the Edmonton Frail Scale related to self-perceived social support ^14^.

Additionally, harmful habits such as smoking and alcohol consumption were included. Smoking was evaluated using a question from the Global Adult Tobacco Survey (GATS), considering as a smoker anyone who reported consuming at least one cigarette per week ^15^. Alcohol consumption was evaluated using the abbreviated version of the Alcohol Use Disorders Identification Test (AUDIT-C), considering cutoff points ≥4 in men and ≥3 in women ^16^.

Sensory problems were also evaluated, including auditory health through the whisper test and self-reported visual health, following the recommendations of the Integrated Care for Older People (ICOPE) manual ^(^^17^.

Finally, geriatric syndromes and comorbidities were included. Depression was assessed using the Yesavage scale, considering the presence of depressive symptoms with scores ≥2 points ^18^. Subjective memory complaint was assessed using the question: "Do you think you have more memory problems than most people?", previously used in research on cognitive function ^19^.

Multimorbidity was defined as the presence of two or more chronic diseases among the following conditions: myocardial infarction, stroke, chronic pain, hypertension, type 2 diabetes mellitus, chronic obstructive pulmonary disease, cancer, congestive heart failure, asthma, arthritis, chronic kidney disease, dengue, Chagas disease, hepatitis B, leishmaniasis, malaria, syphilis, and COVID-19 ^20^.

Dependence in instrumental activities of daily living was evaluated using the Lawton and Brody index, considering dependence scores ≤7 in women and ≤4 in men ^21^. Nutritional problems were evaluated using the Mini Nutritional Assessment (MNA), considering risk of malnutrition or established malnutrition a score ≤24 points ^22^. Frailty was evaluated using the FRAIL scale, which considers exhaustion, fatigue, resistance, illnesses, and weight loss. Subsequently, participants were classified as robust (0 points), pre-frail (1-2 points), and frail (3-5 points) ^(^^23^.

### Statistical analysis

Information from the Amazon Frail study database was exported and analyzed using Stata software for Windows, version 18.0 (StataCorp, College Station, TX, USA). Categorical variables were described using absolute frequencies and percentages. For bivariate analysis, the chi-squared test was used to identify associations between sarcopenia and the other study variables. When expected frequencies in the cells were less than 20, Fisher's exact test was used.

Subsequently, prevalence ratios (PR) and 95% confidence intervals (95% CI) were estimated using Poisson regression models with robust variance. The adjusted models included all study covariates because they are considered confounders according to the literature. Multicollinearity was evaluated using the variance inflation factor (VIF), and no collinearity problems justifying the exclusion of variables were identified. The variable sex was excluded because it is part of the definition of covariates such as: alcohol consumption, sarcopenia, and dependence for instrumental activities.

### Ethical aspects

The original study was approved by the Ethics Committee of the Universidad Científica del Sur (040-2022-PRO99). All participants signed an informed consent prior to their participation. The database used for the present analysis was anonymized prior to its use, guaranteeing the confidentiality and privacy of the information.

## RESULTS

Of the total of 315 study participants, women predominated 61.3% (n=193). Of the participants, 62.4% (n=194) were married; 86.7% (n=273) were illiterate or had completed at least primary school; and 68.3% (n=213) had social support. Furthermore, 93.3% (n=294) did not smoke; 58.33% (n=168) had alcohol consumption risk; 31.1% (n=97) reported visual problems; and 47.2% (n=144) had auditory problems. Regarding geriatric syndromes, 37.5% (n=118) presented depressive symptoms; 29.9% (n=92) reported memory problems; 71.7% (n=226) had one or no comorbidity; 40.6% (n=128) presented functional dependence for IADL; 83.2% (n=262) had nutritional problems; and 38.4% (n=121) were frail. In the evaluation of sarcopenia components, 46.7% (n=147) of the participants presented low muscle mass, 58.1% (n=183) presented decreased muscle strength, and 35.6% (n=112) showed low physical performance, according to the criteria established by the AWGS. Likewise, the global prevalence of sarcopenia in the studied population was 35.9% (n=113) (table 1).

**Table 1:** Descriptive analysis of the study variables (n=315)

Variables		N*	%
Sex			
	Female	193	61.3
	Male	122	38.7
Age			
	60 - 79 years	254	82.5
	≥ 80 years	54	17.5
Marital status			
	Single	82	26.4
	Married/Cohabiting	194	62.4
	Divorced/separated/widowed	35	11.2
Level of education			
	Illiterate, incomplete primary education or complete primary education	273	86.7
	Incomplete secondary, complete secondary, technical, or higher education	42	13.3
Self-report of social support			
	Adequate	213	68.3
	Social risk	99	31.7
Cigarettes per week			
	None	294	93.3
	At least one	21	6.7
Consumption of Alcohol			
	Without risk	147	46.7
	With risk	168	53.3
Self-report of visual problems			
	No	215	68.9
	Yes	97	31.1
Whisper test (hearing problems)			
	Negative	161	52.8
	Positive	144	47.2
Depressive symptoms			
	No	197	62.5
	Yes	118	37.5
Subjective memory complaint			
	No	216	70.1
	Yes	92	29.9
Multimorbidity			
	No	226	71.8
	Yes	89	28.2
Dependence for instrumental activities			
	No	187	59.4
	Yes	128	40.6
Nutritional Problems			
	No	53	16.8
	Yes	262	83.2
	Fragility		
	No	109	34.6
	Pre-Frail	85	27.0
	Fragile	121	38.4
Sarcopenia			
	No	202	64.1
	Yes	113	35.9
Dependence for basic activities			
	No	200	63.5
	Yes	115	36.5

* The size could be different from 315 due to missing data; however, no variable exceeded 10%.

In the bivariate analysis, a statistically significant association was found between functional dependence and age (p<0.001). Among participants with functional dependence, 33.9% were 80 years or older (n=38), compared to 8.2% of those who presented functional independence (n=16). Likewise, a higher proportion of frailty was observed among those with functional dependence, where 63.4% presented frailty (n=73), compared to 24.0% among those who presented functional independence, finding a statistically significant association (p<0.001). Significant associations were also found with hearing problems (p<0.001), depressive symptoms (p=0.002), subjective memory complaint (p<0.001), dependence for instrumental activities (p<0.001), and nutritional problems (p=0.006). Regarding sarcopenia, no statistically significant association was found with functional dependence (p=0.059), although it was more frequent among those who presented functional dependence (42.6%; n=49) than among those who presented functional independence (32.0%; n=64) (table 2).

**Table 2:** Bivariate analysis between the study variables and functional dependence (n=315)

Variables		Functional Independence	Functional dependence	p-value
		N (%)	N (%)	
Sex				
	Female	126 (63.0)	67 (58.3)	0.406
	Male	74 (37.0)	48 (41.7)	
Age^a^				
	60 - 79 years	180 (91.8)	74 (66.1)	<0.001
	≥ 80 years	16 (8.2)	38 (33.9)	
Marital status^a^				
	Single	57 (28.9)	25 (21.9)	0.400
	Married or cohabiting	118 (59.9)	76 (66.7)	
	Divorced, separated or widowed	22 (11.2)	13 (11.4)	
Educational attainment				
	Illiterate, incomplete primary or complete primary	168 (84.0)	105 (91.3)	0.045
	Incomplete secondary, complete, technical or higher	32 (16.0)	10 (8.7)	
Self-report of social support				
	Adequate	138 (69.7)	75 (65.7)	0.475
	Social risk	60 (30.3)	39 (34.2)	
Cigarettes per week^a^				
	None	190 (95.0)	104 (90.5)	0.093
	At least one	10 (5.0)	11 (9.5)	
Alcohol Consumption				
	No risk	97 (48.5)	50 (43.5)	0.390
	With risk	103 (51.5)	65 (56.5)	
Self-report of visual problems				
	No	141 (71.2)	74 (64.9)	0.247
	Yes	57 (28.8)	40 (35.1)	
Whisper test (hearing problems)				
	Negative	123 (63.7)	38 (33.9)	<0.001
	Positive	70 (36.3)	74 (66.1)	
Depressive Symptoms				
	No	138 (69.0)	59 (51.3)	0.002
	Yes	62 (31.0)	56 (48.7)	
Subjective memory complaint				
	No	157 (79.3)	59 (53.6)	<0.001
	Yes	41 (20.7)	51 (46.4)	
Multimorbidity				
	No	147 (73.5)	79 (68.7)	0.362
	Yes	53 (26.5)	36 (31.3)	
Dependency for instrumental activities				
	No	144 (72.0)	43 (37.4)	<0.001
	Yes	56 (28.0)	72 (62.6)	
Nutritional problems^a^				
	No	42 (21.0)	11 (9.6)	0.006
	Yes	158 (79.0)	104 (90.4)	
Frailty				
	No	88 (44.0)	21 (18.3)	<0.001
	Pre-Frailty	64 (32.0)	21 (18.3)	
	Fragile	48 (24.0)	73 (63.4)	
Sarcopenia				
	No	136 (68.0)	66 (57.3)	0.059
	Yes	64 (32.0)	49 (42.6)	

a p values were calculated using the chi^2^ test; if the expected frequencies in the cells were less than 20, Fisher's exact test was used.

In Table 3, we can observe an adjusted Poisson regression analysis to quantify the association between sarcopenia and functional dependence. We found that in a model adjusted for depressive symptoms, risk of alcohol consumption, subjective memory complaints, age, marital status, educational level, multimorbidity, harmful habits, smoking, sensory problem, frailty, malnutrition, and dependence for Instrumental Activities of Daily Living (IADL) for daily life, having sarcopenia is associated with a 1.43 times higher probability of having functional dependence.

**Table 3:** Association between sarcopenia and functional dependence in basic activities of daily living in older adults from rural communities of the Peruvian Amazon (n = 315)

Variable		Crude RP (95% CI)^a^	p-value	adjusted PR (95% CI)^b^	p-value
Sarcopenia					
	No	References	0.055	References	0.024
	Yes	1.32 (0.99 - 1.77)		1.43 (1.05 - 1.95)	

a PR: prevalence ratio; 95%CI: 95% confidence interval. ^b^The adjusted model was estimated using Poisson regression with robust variance and was adjusted for age, marital status, educational level, social support, smoking, alcohol consumption, visual problems, hearing problems, depressive symptoms, subjective memory complaint, multimorbidity, dependence for instrumental activities, nutritional problems, and frailty.

## DISCUSSION

The present study showed that the presence of sarcopenia was associated with a higher probability of dependence for basic activities of daily living, even after adjusting for sociodemographic, clinical, and geriatric variables. This finding differs from that reported in rural communities in Brazil, where no significant association was found between sarcopenia and functional dependence, attributing a greater role to physical performance, cerebrovascular disease, and age ^24^. These discrepancies could be explained by methodological differences between the studies, particularly in the characteristics of the included populations, the instruments used to assess functionality, and the criteria used to define sarcopenia. Furthermore, our population consisted exclusively of older adults residing in Amazonian rural communities, a context characterized by limitations in access to health services and a high burden of geriatric syndromes, factors that could modify the relationship between sarcopenia and functionality.

On the contrary, our results are consistent with the findings of a Colombian national survey on aging, in which it was observed that functional dependence was associated with the presence of sarcopenia, even after adjusting for potential confounding factors ^25^. The concordance between both studies reinforces the hypothesis that sarcopenia constitutes an important determinant of functional decline in older people.

The concordance between both studies reinforces the hypothesis that sarcopenia constitutes an important determinant of functional decline in older adults. From a biological perspective, this association is plausible, since sarcopenia is characterized by a progressive decrease in muscle mass, strength, and physical performance, essential components for the execution of basic activities of daily living ^13^. The loss of muscle mass and strength reduces the ability to perform tasks requiring mobility, balance, and coordination, such as bathing, dressing, transferring, or eating, thus favoring the appearance of functional limitations and dependence ^26^. Furthermore, sarcopenia has been related to neuromuscular alterations, chronic low-grade inflammation, oxidative stress, and decreased physiological reserve, mechanisms that contribute to the progressive deterioration of functionality and a reduced capacity to respond to acute stress situations ^27^^,^^28^.

Various longitudinal studies have shown that older adults with sarcopenia have a higher risk of developing incident disability in both basic and instrumental activities of daily living compared to those without sarcopenia ^26^^,^^28^. Likewise, the decline in specific components of sarcopenia, particularly grip strength and gait speed, has been associated with persistent trajectories of functional disability and long-term loss of independence ^29^. These findings suggest that sarcopenia not only constitutes a marker of biological aging but also an important predictor of adverse outcomes related to functionality.

Additionally, a study conducted in India evidenced contextual differences in factors related to sarcopenia between rural and urban populations ^30^. While, in rural areas, sarcopenia was mainly associated with age and socioeconomic status, in urban settings it was related to age and sedentary lifestyle ^30^. These findings suggest that sarcopenia prevention and management strategies should be adapted to the particular characteristics of each context. Likewise, the clinical relevance of sarcopenia transcends the functional domain. It has been documented that muscle alterations evaluated by the Timed Up and Go test are associated with higher mortality in older adults after oncological surgeries ^31^. Taken together, this evidence highlights the need to timely identify and address sarcopenia, particularly in vulnerable rural populations. In this regard, early detection and the development of multimodal interventions based on physical exercise and nutritional support could contribute to preventing functional decline and improving the quality of life of older adults ^32^.

Various international guidelines recommend that the approach to sarcopenia be based on early detection, periodic assessment of functional status, and the implementation of multimodal interventions, particularly resistance exercise and optimization of nutritional status ^33^. Consistently, a recent systematic review and meta-analysis showed that exercise-based interventions in older adults with sarcopenia significantly improve lower limb muscle strength and gait speed, thus contributing to preserving functionality and independence ^34^. These findings reinforce the need to incorporate sarcopenia screening and timely management strategies in primary care, especially in vulnerable rural populations.

This research presents some limitations. First, due to its cross-sectional design, it is not possible to establish causal relationships between the analyzed variables nor to determine the temporal directionality of the observed associations. Second, muscle mass was estimated using single-frequency bioelectrical impedance (50 kHz), while the guidelines of the Asian Working Group for Sarcopenia preferably recommend the use of multi-frequency equipment, which could have generated errors in the classification of sarcopenia. Likewise, functional dependence was defined using a Barthel index cutoff point previously employed in a population with stroke, so its applicability to older adults from Amazonian rural communities could be limited. Additionally, as it was a secondary data analysis, the study was restricted to the variables available in the original database, which limited the possibility of incorporating other potential confounding factors or thoroughly verifying all procedures related to data collection. Although adjusted for multiple clinically relevant covariates, the presence of residual confounding due to unmeasured variables cannot be ruled out.

On the other hand, the sample presented a higher proportion of women, which could limit the extrapolation of the findings to populations with a more balanced sex distribution. Likewise, the original study used non-probabilistic sampling, which restricts the representativeness of the sample and prevents completely ruling out the presence of selection bias. Consequently, the results should be interpreted with caution and cannot be generalized to all older adults residing in the Peruvian Amazon. Future research should consider longitudinal designs, probabilistic samples, and more representative populations to confirm the observed findings.

Nevertheless, the study presents important strengths. The diagnosis of sarcopenia was performed by highly trained personnel, with clinical and research experience, using validated instruments and equipment that allow for precise evaluation. Furthermore, although the association between sarcopenia and functionality has been previously studied, rural communities, particularly those located in jungle regions, have been scarcely explored. Providing evidence from these contexts contributes to decision-making based on real population needs, which may differ according to sociodemographic, economic, and geographic characteristics.

In conclusion, sarcopenia is associated with an increase in the probability of presenting functional dependence for basic activities of daily living. We recommend that, in addition to routine functional assessment in rural communities, sarcopenia be incorporated into geriatric syndrome screening, given its relationship with various adverse health outcomes, including functional dependence. Early identification would allow for timely interventions and promote geriatric care focused on individual needs, aimed at preserving autonomy and improving the quality of life of older adults.
