# Supplementary material for: Association between sarcopenia and functionality in older adults in the Peruvian Amazon
Source: Rev Peru Med Exp Salud Publica. 2026 Sep 29;43(3):330–7. doi: 10.17843/rpmesp.2026.433.16113 (PMC13718561; doi:10.17843/rpmesp.2026.433.16113)
Supplement: Supplementary file 2 [file rpmesp-43-03-16113-s001.docx]

**Material suplementario**

**Figura**. Diagrama acíclico dirigido (DAG) con las variables potencialmente confusoras consideradas en el análisis.
