# Supplementary material for: Association between sarcopenia and functionality in older adults in the Peruvian Amazon
Source: Rev Peru Med Exp Salud Publica. 2026 Sep 29;43(3):330–7. doi: 10.17843/rpmesp.2026.433.16113 (PMC13718561; doi:10.17843/rpmesp.2026.433.16113)
Supplement: Translation

# Translation: Asociación entre sarcopenia y funcionalidad en personas mayores de la Amazonía peruana

**Authors:** Fabrizio Carlos-Mallorga, Jarumi M. Flores-Samamé, Naskia L. Valdivia-Salas, Alvaro M. Ñaña-Cordova, Xiomara C. Benavente-Chalco, Kiara Camacho-Caballero, José F. Parodi, Fernando M. Runzer-Colmenares

**Affiliations:** 1 Carrera de Medicina Humana, Universidad Científica del Sur. Lima, Perú.; 2 CHANGE Research Working Group, Facultad de Ciencias de la Salud, Universidad Científica del Sur. Lima, Perú.; 3 Centro de Investigación del Envejecimiento (CIEN), Facultad de Medicina Humana, Universidad de San Martín de Porres. Lima, Perú.

**Keywords:** Sarcopenia, Estado Funcional, Fuerza Muscular, Envejecimiento, Amazonía

## Abstract

**Objetivos.:**

Analizar la relación entre sarcopenia y la dependencia funcional en las personas mayores de la Amazonía peruana.

**Materiales y métodos.:**

Se realizó un estudio transversal basado en un análisis secundario de datos del estudio Amazon Frail que incluyó a personas mayores (60 años o más). Se evaluó sarcopenia mediante los criterios establecidos por el Asian Working Group for Sarcopenia que incluyen la medición de fuerza muscular, masa muscular y rendimiento físico; y la funcionalidad, mediante la escala de Barthel. Además, se incluyeron características sociodemográficas y antropométricas, antecedentes médicos y personales. Se realizó un análisis de regresión de Poisson con varianza robusta para estimar razones de prevalencia (RP) crudas y ajustadas, con sus respectivos intervalos de confianza al 95% (IC95%).

**Resultados.:**

Se incluyeron en el análisis a 315 personas mayores, de los cuales, 193 (n=61,3%) eran mujeres. Se halló que 113 (n=35,9%) presentaban sarcopenia y 115 (n=36,5%) tenían dependencia funcional. En el análisis de regresión de Poisson ajustado, se encontró que la sarcopenia se asoció significativamente con mayor probabilidad de dependencia funcional (RP=1,43; IC95%: 1,05-1,95). Además, la fragilidad (RP=2,07; IC95%: 1,27-3,38), la queja subjetiva de memoria (RP=1,40; IC95%: 1,02-1,91), y la pérdida auditiva (RP=1,53; IC95%: 1,09-2,14) también fueron factores asociados a mayor dependencia funcional.

**Conclusiones.:**

En comunidades amazónicas rurales, la sarcopenia en personas mayores está asociada a dependencia funcional. Su identificación temprana es fundamental para prevenir la dependencia funcional y orientar intervenciones integrales en salud.

## INTRODUCCIÓN

El aumento sostenido de la esperanza de vida ha orientado la atención hacia el concepto de envejecimiento saludable, entendido como la preservación de la funcionalidad en las personas mayores ^1^. La funcionalidad se define como la capacidad para vivir de manera independiente, realizar actividades básicas y asumir el autocuidado, siendo la dependencia en actividades básicas de la vida diaria (ABVD) una de las manifestaciones más severas del deterioro funcional en las personas mayores. ^1^. A nivel internacional, se han reportado prevalencias de dependencia moderada y severa de 10,2% y 4,8%hacia las actividades de la vida diaria, respectivamente ^2^. No obstante, la alteración de la funcionalidad no es homogénea y varía según la carga de comorbilidad y el contexto de atención. Por ejemplo, en zonas rurales altoandinas del Perú se ha documentado una prevalencia de dependencia funcional del 51,1% en actividades básicas de la vida diaria en personas mayores, cifra que anticipa una elevada demanda de recursos sanitarios en el mediano plazo ^3^. Este hallazgo adquiere relevancia adicional si se considera que la dependencia funcional se ha asociado con mayor mortalidad hospitalaria en esta población ^4^. Aunque la dependencia funcional suele ser un proceso gradual y progresivo, la aparición de condiciones médicas que conducen a la dependencia en el adulto mayor continúa representando un desafío prioritario de salud pública.

Entre las condiciones estrechamente vinculadas al envejecimiento se encuentra la sarcopenia, un síndrome geriátrico multifactorial caracterizado por la pérdida progresiva y generalizada de masa muscular, acompañada de disminución de la fuerza y del rendimiento físico ^1^^,^^5^ Esta condición incrementa la vulnerabilidad y el riesgo de dependencia en la persona mayor cuando no se identifica e interviene oportunamente ^1^. Su prevalencia aumenta marcadamente con la edad, oscilando entre 5% y 13% en adultos de 60 a 70 años y superando el 50% en aquellos mayores de 80 años ^5^. En el contexto del incremento de la esperanza de vida, es previsible que la carga de sarcopenia también se eleve, dado que la fuerza muscular comienza a declinar desde la tercera década de la vida. Ello complejiza su abordaje clínico y constituye un desafío relevante por su impacto directo sobre la funcionalidad y la autonomía de las personas mayores ^6^.

Dado que la sarcopenia puede acelerar la aparición de dependencia funcional, resulta fundamental evaluar sus posibles asociaciones considerando las particularidades demográficas, culturales y clínicas de cada población. En Perú, un estudio hospitalario reportó una prevalencia de sarcopenia del 49,4%, lo que evidencia la magnitud del problema en el ámbito clínico ^7^. Asimismo, se ha descrito que la presencia de sarcopenia incrementa en 1,58 veces el riesgo de desarrollar dependencia funcional ^8^. No obstante, la evidencia disponible sobre sarcopenia y deterioro funcional en personas mayores residentes en áreas rurales continúa siendo limitada. La mayoría de las investigaciones nacionales se han centrado en poblaciones rurales andinas, en las cuales se ha documentado una prevalencia de sarcopenia del 17,6% y una asociación significativa entre esta condición y la dependencia funcional ^9^.

La población geriátrica de la Amazonía peruana, pese a presentar características ambientales, socioculturales y nutricionales particulares, sigue siendo escasamente explorada en la literatura científica. En este contexto, el objetivo del presente estudio fue analizar la relación entre la sarcopenia y la dependencia funcionalidad en personas mayores residentes en la Amazonía peruana.

**MENSAJES CLAVE**

Motivación del estudio. En la Amazonía peruana, muchas personas mayores viven en comunidades rurales, con desplazamientos difíciles, y limitado acceso a servicios de salud. En este contexto, se conoce poco sobre cómo la sarcopenia afecta su vida diaria.

Principales hallazgos. Evaluamos adultos mayores que viven en comunidades amazónicas rurales y encontramos que la sarcopenia fue frecuente. Quienes la presentaban tenían mayor probabilidad de necesitar ayuda para bañarse, vestirse o movilizarse.

Implicancias en salud pública. Es fundamental detectar la sarcopenia de manera temprana. Se requieren programas comunitarios de ejercicio, nutrición y apoyo familiar, adaptados a la realidad geográfica y cultural amazónica, para proteger la autonomía y la calidad de vida de las personas mayores.

## MATERIALES Y MÉTODOS

### Diseño y contexto del estudio

Se realizó un estudio observacional, analítico, transversal y retrospectivo, correspondiente a un análisis secundario de datos provenientes del estudio Amazon Frail ^10^. Los datos fueron recolectados entre marzo y julio de 2024 en comunidades rurales de la Amazonía peruana. El estudio original tuvo como objetivo evaluar el estado de fragilidad y dependencia funcional en personas mayores residentes en dichas comunidades. Los participantes fueron seleccionados mediante un muestreo no probabilístico de tipo censal e incluyó a personas de 60 años o más residentes en las comunidades evaluadas ^10^. El presente manuscrito fue elaborado siguiendo las recomendaciones de la guía Strengthening the Reporting of Observational Studies in Epidemiology (STROBE) para estudios observacionales transversales.

### Participantes

Para el estudio original, se incluyeron personas de 60 años o más y se excluyeron participantes que presentaron deterioro cognitivo moderado o severo, definido mediante ocho o más errores en el cuestionario de Pfeiffer, déficit visual o auditivo que limitara la realización de las evaluaciones físicas o la entrevista, rechazo a participar, o dificultades para expresarse o comprender el idioma español ^10^.

Para el presente análisis secundario, se incluyeron únicamente los registros que contaban con información completa sobre las variables de interés sarcopenia y funcionalidad. Se excluyeron los registros con datos incompletos en cualquiera de las variables principales. No se identificaron datos faltantes en las variables de interés, por lo que todos los registros elegibles fueron incluidos en el análisis final, alcanzándose una muestra de 315 participantes.

### Variables principales

La variable desenlace fue la dependencia en actividades básicas de la vida diaria (ABVD), evaluada mediante el índice de Barthel. Este instrumento valora la capacidad funcional para realizar 10 actividades básicas de la vida diaria, con puntuaciones que oscilan entre 0 y 100 puntos ^11^. Los participantes con puntajes ≤80 fueron clasificados como dependientes funcionales, mientras que aquellos con puntajes >80 fueron considerados funcionalmente independientes ^11^.

La variable exposición fue la sarcopenia, evaluada siguiendo los criterios diagnósticos propuestos por el Asian Working Group for Sarcopenia 2019 (AWGS) ^(^^12^, los cuales consideran la evaluación de la fuerza muscular, la masa muscular y el rendimiento físico.

La fuerza muscular fue evaluada mediante la medición de la fuerza de prensión manual de la mano dominante utilizando un dinamómetro hidráulico Jamar®. Se consideró fuerza muscular disminuida un valor ≤28 kg en hombres y ≤18 kg en mujeres, de acuerdo con los puntos de corte establecidos por el AWGS ^12^.

La masa muscular fue estimada mediante análisis de bioimpedancia eléctrica (BIA) utilizando el equipo BodyStat®500 (Bodystat LTD, Douglas, Isle of Man, UK), de monofrecuencia (50 kHz), con el participante en posición de decúbito dorsal. La masa muscular esquelética apendicular (ASMM) se estimó utilizando la siguiente ecuación: ASMM (kg) = -0,05376 + (0,2394 × H²/R) + (2,708 × sexo) + (0,065 × peso) donde H corresponde a la talla (cm), R a la resistencia medida a 50 kHz y sexo se codificó como 1 para hombres y 0 para mujeres. Posteriormente, se calculó el índice de masa muscular esquelética (SMI = ASMM/talla²), considerándose masa muscular baja valores ≤7,0 kg/m² en hombres y ≤5,7 kg/m² en mujeres ^13^.

El rendimiento físico fue evaluado mediante la batería corta de desempeño físico (Short Physical Performance Battery, SPPB), que incluye pruebas de equilibrio, velocidad de marcha en 4 metros y levantarse de una silla. Se consideró bajo rendimiento físico un puntaje ≤9 puntos ^12^.

Se clasificó como sarcopenia a la presencia simultánea de disminución de la fuerza muscular y masa muscular (sarcopenia confirmada), así como a la presencia concomitante de disminución de la fuerza muscular, masa muscular y rendimiento físico (sarcopenia severa). Para el análisis, ambas categorías fueron agrupadas en una única variable dicotómica (presencia/ausencia de sarcopenia).

### Covariables

La identificación del conjunto de covariables se realizó mediante un diagrama acíclico dirigido (DAG) (material suplementario).

Se incluyeron variables sociodemográficas como sexo (masculino/femenino), edad (años), estado civil (soltero, casado/conviviente, divorciado/separado/viudo), grado de instrucción (analfabeto/primaria incompleta/primaria completa/secundaria incompleta/secundaria completa/educación técnica/educación superior) y el apoyo social, evaluado mediante una pregunta del cuestionario de fragilidad de Edmonton relacionada con el soporte social autopercibido ^14^.

Asimismo, se incluyeron hábitos nocivos como tabaquismo y consumo de alcohol. El tabaquismo fue evaluado mediante una pregunta de la Encuesta Mundial de Tabaquismo en Adultos (GATS), considerando fumador a quien reportó consumir al menos un cigarrillo por semana ^15^. El consumo de alcohol fue evaluado mediante la versión abreviada del Alcohol Use Disorders Identification Test (AUDIT-C), considerando puntos de corte ≥4 en hombres y ≥3 en mujeres ^16^.

También se evaluaron problemas sensoriales, incluyendo salud auditiva mediante la prueba del susurro y salud visual autorreportada, siguiendo las recomendaciones del manual Integrated Care for Older People (ICOPE) ^(^^17^.

Finalmente, se incluyeron síndromes geriátricos y comorbilidades. La depresión fue evaluada mediante la escala de Yesavage, considerándose la presencia de síntomas depresivos con puntuaciones ≥2 puntos ^18^. La queja subjetiva de memoria fue evaluada mediante la pregunta: “¿Cree que tiene más problemas de memoria que la mayoría de la gente?”, utilizada previamente en investigaciones sobre función cognitiva ^19^.

La multimorbilidad fue definida como la presencia de dos o más enfermedades crónicas entre las siguientes condiciones: infarto de miocardio, accidente cerebrovascular, dolor crónico, hipertensión arterial, diabetes mellitus tipo 2, enfermedad pulmonar obstructiva crónica, cáncer, insuficiencia cardíaca congestiva, asma, artritis, enfermedad renal crónica, dengue, enfermedad de Chagas, hepatitis B, leishmaniasis, malaria, sífilis y COVID-19 ^20^.

La dependencia en actividades instrumentales de la vida diaria fue evaluada mediante el índice de Lawton y Brody, considerando dependencia puntajes ≤7 en mujeres y ≤4 en hombres ^21^. Los problemas nutricionales fueron evaluados mediante el Mini Nutritional Assessment (MNA), considerándose riesgo de malnutrición o malnutrición establecida un puntaje ≤24 puntos ^22^. La fragilidad se evaluó mediante la escala FRAIL, la cual considera agotamiento, fatiga, resistencia, enfermedades y pérdida de peso. Posteriormente, los participantes fueron clasificados como robustos (0 puntos), prefrágiles (1-2 puntos) y frágiles (3-5 puntos) ^(^^23^.

### Análisis estadístico

La información proveniente de la base de datos del estudio Amazon Frail fue exportada y analizada utilizando el software Stata para Windows, versión 18.0 (StataCorp, College Station, TX, USA). Las variables categóricas fueron descritas mediante frecuencias absolutas y porcentajes. Para el análisis bivariado se empleó la prueba de chi cuadrado con el objetivo de identificar asociaciones entre la sarcopenia y las demás variables del estudio. Cuando las frecuencias esperadas en las celdas fueron menores de 20, se utilizó la prueba exacta de Fisher.

Posteriormente, se estimaron razones de prevalencia (RP) e intervalos de confianza al 95% (IC95%) mediante modelos de regresión de Poisson con varianza robusta. Los modelos ajustados incluyeron todas las covariables del estudio porque son consideradas como confusoras según la literatura. La multicolinealidad fue evaluada mediante el factor de inflación de la varianza (VIF), sin identificarse problemas de colinealidad que justificaran la exclusión de variables. Se excluyó a la variable sexo debido a que forma parte de la definición de covariables como: consumo de alcohol, sarcopenia y dependencia para actividades instrumentales.

### Aspectos éticos

El estudio original fue aprobado por el Comité de Ética de la Universidad Científica del Sur (040-2022-PRO99). Todos los participantes firmaron un consentimiento informado previo a su participación. La base de datos utilizada para el presente análisis fue anonimizada previamente a su uso, garantizando la confidencialidad y privacidad de la información.

## RESULTADOS

Del total de 315 participantes del estudio, predominaron las mujeres 61,3% (n=193). De los participantes, eran casados 62,4% (n=194); analfabetos o habían cursado al menos la primaria 86,7% (n=273); y contaban con apoyo social 68,3% (n=213). Además, no fumaban 93,3% (n=294); tuvieron riesgo por consumo de alcohol 58.33% (n=168); reportaron problemas visuales 31,1% (n=97); y se encontraron problemas auditivos 47,2% (n=144). Con respecto a los síndromes geriátricos, presentaron síntomas depresivos 37,5% (n=118); reportaron problemas de memoria 29,9% (n=92); tuvieron una o ninguna comorbilidad 71,7% (n=226); presentaron dependencia funcional para AIVD 40,6% (n=128); tuvieron problemas nutricionales 83,2% (n=262); y fueron frágiles 38,4% (n=121). En la evaluación de los componentes de la sarcopenia, el 46,7% (n=147) de los participantes presentó baja masa muscular, el 58,1% (n=183) presentó disminución de la fuerza muscular y el 35,6% (n=112) evidenció bajo rendimiento físico, de acuerdo con los criterios establecidos por el AWGS. Asimismo, la prevalencia global de sarcopenia en la población estudiada fue de 35,9% (n=113) (tabla 1).

**Tabla 1:** Análisis descriptivo de las variables de estudio (n=315)

Variables		N*	%
Sexo			
	Femenino	193	61,3
	Masculino	122	38,7
Edad			
	60 - 79 años	254	82,5
	≥ 80 años	54	17,5
Estado civil			
	Soltero	82	26,4
	Casado/conviviente	194	62,4
	Divorciado/separado/ viudo	35	11,2
Grado de instrucción			
	Analfabeto, primaria incompleta o primaria completa	273	86,7
	Secundaria incompleta, completa, técnico o superior	42	13,3
Autorreporte de apoyo social			
	Adecuado	213	68,3
	Riesgo social	99	31,7
Cigarrillos por semana			
	Ninguno	294	93,3
	Al menos uno	21	6,7
Consumo de alcohol			
	Sin riesgo	147	46,7
	Con riesgo	168	53,3
Autorreporte de problemas visuales			
	No	215	68,9
	Sí	97	31,1
Test del susurro (problemas auditivos)			
	Negativo	161	52,8
	Positivo	144	47,2
Síntomas depresivos			
	No	197	62,5
	Sí	118	37,5
Queja subjetiva de memoria			
	No	216	70,1
	Sí	92	29,9
Multimorbilidad			
	No	226	71,8
	Sí	89	28,2
Dependencia para actividades instrumentales			
	No	187	59,4
	Sí	128	40,6
Problemas nutricionales			
	No	53	16,8
	Sí	262	83,2
	Fragilidad		
	No	109	34,6
	Pre Frágil	85	27,0
	Frágil	121	38,4
Sarcopenia			
	No	202	64,1
	Sí	113	35,9
Dependencia para actividades básicas			
	No	200	63,5
	Sí	115	36,5

*El tamaño podría ser diferente a 315 debido a datos faltantes, sin embargo, en ninguna variable se excedió del 10%.

En el análisis bivariado se encontró una asociación estadísticamente significativa entre la dependencia funcional y la edad (p<0,001). Entre los participantes con dependencia funcional, el 33,9% tenía 80 años o más (n=38), en comparación con el 8,2% de quienes presentaron independencia funcional (n=16). Asimismo, se observó una mayor proporción de fragilidad entre aquellos con dependencia funcional, donde el 63,4% presentó fragilidad (n=73), frente al 24,0% entre quienes presentaron independencia funcional, encontrándose una asociación estadísticamente significativa (p<0,001). También se encontraron asociaciones significativas con los problemas auditivos (p<0,001), síntomas depresivos (p=0,002), queja subjetiva de memoria (p<0,001), dependencia para actividades instrumentales (p<0,001) y problemas nutricionales (p=0,006). En relación con la sarcopenia, no se encontró una asociación estadísticamente significativa con la dependencia funcional (p=0,059), aunque fue más frecuente entre quienes presentaron dependencia funcional (42,6%; n=49) que entre quienes presentaron independencia funcional (32,0%; n=64) (tabla 2).

**Tabla 2:** Análisis bivariado entre las variables del estudio y dependencia funcional (n=315)

Variables		Independencia funcional	Dependencia funcional	Valor de p
		N (%)	N (%)	
Sexo				
	Femenino	126 (63,0)	67 (58,3)	0,406
	Masculino	74 (37,0)	48 (41,7)	
Edad^a^				
	60 - 79 años	180 (91,8)	74 (66,1)	<0,001
	≥ 80 años	16 (8,2)	38 (33,9)	
Estado civil^a^				
	Soltero	57 (28,9)	25 (21,9)	0,400
	Casado o conviviente	118 (59,9)	76 (66,7)	
	Divorciado, separado o viudo	22 (11,2)	13 (11,4)	
Grado de instrucción				
	Analfabeto, primaria incompleta o primaria completa	168 (84,0)	105 (91,3)	0,045
	Secundaria incompleta, completa, técnico o superior	32 (16,0)	10 (8,7)	
Autorreporte de apoyo social				
	Adecuado	138 (69,7)	75 (65,7)	0,475
	Riesgo social	60 (30,3)	39 (34,2)	
Cigarrillos por semana^a^				
	Ninguno	190 (95,0)	104 (90,5)	0,093
	Al menos uno	10 (5,0)	11 (9,5)	
Consumo de alcohol				
	Sin riesgo	97 (48,5)	50 (43,5)	0,390
	Con riesgo	103 (51,5)	65 (56,5)	
Autorreporte de problemas visuales				
	No	141 (71,2)	74 (64,9)	0,247
	Sí	57 (28,8)	40 (35,1)	
Test del susurro (problemas auditivos)				
	Negativo	123 (63,7)	38 (33,9)	<0,001
	Positivo	70 (36,3)	74 (66,1)	
Síntomas depresivos				
	No	138 (69,0)	59 (51,3)	0,002
	Sí	62 (31,0)	56 (48,7)	
Queja subjetiva de memoria				
	No	157 (79,3)	59 (53,6)	<0,001
	Sí	41 (20,7)	51 (46,4)	
Multimorbilidad				
	No	147 (73,5)	79 (68,7)	0,362
	Sí	53 (26,5)	36 (31,3)	
Dependencia para actividades instrumentales				
	No	144 (72,0)	43 (37,4)	<0,001
	Sí	56 (28,0)	72 (62,6)	
Problemas nutricionales^a^				
	No	42 (21,0)	11 (9,6)	0,006
	Sí	158 (79,0)	104 (90,4)	
Fragilidad				
	No	88 (44,0)	21 (18,3)	<0,001
	Pre Frágil	64 (32,0)	21 (18,3)	
	Frágil	48 (24,0)	73 (63,4)	
Sarcopenia				
	No	136 (68,0)	66 (57,3)	0,059
	Sí	64 (32,0)	49 (42,6)	

a Los valores de p fueron calculados mediante la prueba de chi^2^, en caso las frecuencias esperadas en las celdas fueran menores a 20 se utilizó la prueba exacta de Fisher.

En la tabla 3 podemos apreciar un análisis de regresión de Poisson ajustado para cuantificar la asociación entre sarcopenia y dependencia funcional, encontramos que en un modelo ajustado por síntomas depresivos, riesgo de consumo de alcohol, quejas subjetivas de memoria, edad, estado civil, grado de instrucción, multimorbilidad, hábitos nocivos, fumar, problema sensorial, fragilidad, malnutrición y dependencia para AIVD para la vida diaria, tener sarcopenia se asocia con una probabilidad de 1,43 veces mayor de tener dependencia funcional.

**Tabla 3:** Asociación entre sarcopenia y dependencia funcional en actividades básicas de la vida diaria en personas mayores de comunidades rurales de la Amazonía peruana (n = 315)

Variable		RP crudo (IC 95%)^a^	Valor de p	RP ajustado (IC 95%)^b^	Valor de p
Sarcopenia					
	No	Referencia	0,055	Referencia	0,024
	Sí	1,32 (0,99 - 1,77)		1,43 (1,05 - 1,95)	

a RP: razón de prevalencia; IC95%: intervalo de confianza al 95%. ^b^ El modelo ajustado fue estimado mediante regresión de Poisson con varianza robusta y se ajustó por edad, estado civil, grado de instrucción, apoyo social, tabaquismo, consumo de alcohol, problemas visuales, problemas auditivos, síntomas depresivos, queja subjetiva de memoria, multimorbilidad, dependencia para actividades instrumentales, problemas nutricionales y fragilidad.

## DISCUSIÓN

El presente estudio mostró que la presencia de sarcopenia se asoció con una mayor probabilidad de dependencia para las actividades básicas de la vida diaria, incluso después de ajustar por variables sociodemográficas, clínicas y geriátricas. Este hallazgo difiere de lo reportado en comunidades rurales de Brasil, donde no se encontró una asociación significativa entre sarcopenia y dependencia funcional, atribuyéndose un mayor papel al rendimiento físico, la enfermedad cerebrovascular y la edad ^24^. Estas discrepancias podrían explicarse por diferencias metodológicas entre los estudios, particularmente en las características de las poblaciones incluidas, los instrumentos utilizados para evaluar la funcionalidad y los criterios empleados para definir la sarcopenia. Además, nuestra población estuvo conformada exclusivamente por personas mayores residentes en comunidades rurales amazónicas, un contexto caracterizado por limitaciones en el acceso a servicios de salud y una elevada carga de síndromes geriátricos, factores que podrían modificar la relación entre sarcopenia y funcionalidad.

Por el contrario, nuestros resultados son consistentes con los hallazgos de una encuesta nacional colombiana sobre envejecimiento, en la que se observó que la dependencia funcional se asociaba con la presencia de sarcopenia, incluso después de ajustar por potenciales factores de confusión ^25^. La concordancia entre ambos estudios refuerza la hipótesis de que la sarcopenia constituye un importante determinante del deterioro funcional en las personas mayores.

La concordancia entre ambos estudios refuerza la hipótesis de que la sarcopenia constituye un importante determinante del deterioro funcional en las personas mayores. Desde una perspectiva biológica, esta asociación resulta plausible, ya que la sarcopenia se caracteriza por una disminución progresiva de la masa muscular, la fuerza y el rendimiento físico, componentes esenciales para la ejecución de las actividades básicas de la vida diaria ^13^. La pérdida de masa y fuerza muscular reduce la capacidad para realizar tareas que requieren movilidad, equilibrio y coordinación, tales como bañarse, vestirse, trasladarse o alimentarse, favoreciendo así la aparición de limitaciones funcionales y dependencia ^26^. Además, la sarcopenia se ha relacionado con alteraciones neuromusculares, inflamación crónica de bajo grado, estrés oxidativo y disminución de la reserva fisiológica, mecanismos que contribuyen al deterioro progresivo de la funcionalidad y a una menor capacidad para responder a situaciones de estrés agudo ^27^^,^^28^.

Diversos estudios longitudinales han demostrado que las personas mayores con sarcopenia presentan un mayor riesgo de desarrollar discapacidad incidente tanto en actividades básicas como instrumentales de la vida diaria en comparación con aquellas sin sarcopenia ^26^^,^^28^. Asimismo, la disminución de componentes específicos de la sarcopenia, particularmente la fuerza de prensión y la velocidad de la marcha, se ha asociado con trayectorias persistentes de discapacidad funcional y pérdida de independencia a largo plazo ^29^. Estos hallazgos sugieren que la sarcopenia no solo constituye un marcador de envejecimiento biológico, sino también un importante predictor de resultados adversos relacionados con la funcionalidad.

Adicionalmente, un estudio realizado en la India evidenció diferencias contextuales en los factores relacionados con la sarcopenia entre poblaciones rurales y urbanas ^30^. Mientras que, en áreas rurales, la sarcopenia se asoció principalmente con la edad y el nivel socioeconómico, en entornos urbanos se relacionó con la edad y el sedentarismo ^30^. Estos hallazgos sugieren que las estrategias de prevención y manejo de la sarcopenia deberían adaptarse a las características particulares de cada contexto. Asimismo, la relevancia clínica de la sarcopenia trasciende el ámbito funcional. Se ha documentado que las alteraciones musculares evaluadas mediante la prueba Timed Up and Go se asocian con una mayor mortalidad en personas mayores despues de cirugias oncologicas ^31^. En conjunto, esta evidencia destaca la necesidad de identificar y abordar oportunamente la sarcopenia, particularmente en poblaciones rurales vulnerables. En este sentido, la detección temprana y el desarrollo de intervenciones multimodales basadas en ejercicio físico y soporte nutricional podrían contribuir a prevenir el deterioro funcional y mejorar la calidad de vida de las personas mayores ^32^.

Diversas guías internacionales recomiendan que el abordaje de la sarcopenia se base en la detección temprana, la evaluación periódica del estado funcional y la implementación de intervenciones multimodales, particularmente ejercicio de resistencia y optimización del estado nutricional ^33^. De manera consistente, una revisión sistemática y metaanálisis reciente mostró que las intervenciones basadas en ejercicio en personas mayores con sarcopenia mejoran significativamente la fuerza muscular de extremidades inferiores y la velocidad de marcha, contribuyendo así a preservar la funcionalidad y la independencia ^34^. Estos hallazgos refuerzan la necesidad de incorporar estrategias de tamizaje y manejo oportuno de la sarcopenia en el primer nivel de atención, especialmente en poblaciones rurales vulnerables.

Esta investigación presenta algunas limitaciones. En primer lugar, debido a su diseño transversal, no es posible establecer relaciones causales entre las variables analizadas ni determinar la direccionalidad temporal de las asociaciones observadas. En segundo lugar, la masa muscular fue estimada mediante bioimpedancia eléctrica monofrecuencia (50 kHz), mientras que las guías del Asian Working Group for Sarcopenia recomiendan preferentemente el uso de equipos multifrecuencia, lo que podría haber generado errores en la clasificación de la sarcopenia. Asimismo, la dependencia funcional fue definida utilizando un punto de corte del índice de Barthel previamente empleado en una población con accidente cerebrovascular, por lo que su aplicabilidad a personas mayores de comunidades rurales amazónicas podría ser limitada. Adicionalmente, al tratarse de un análisis secundario de datos, el estudio estuvo restringido a las variables disponibles en la base de datos original, lo que limitó la posibilidad de incorporar otros potenciales factores de confusión o verificar detalladamente todos los procedimientos relacionados con la recolección de la información. Aunque se ajustó por múltiples covariables clínicamente relevantes, no puede descartarse la presencia de confusión residual debida a variables no medidas.

Por otro lado, la muestra presentó una mayor proporción de mujeres, lo que podría limitar la extrapolación de los hallazgos a poblaciones con una distribución más equilibrada por sexo. Asimismo, el estudio original utilizó un muestreo no probabilístico, lo cual restringe la representatividad de la muestra e impide descartar completamente la presencia de sesgo de selección. En consecuencia, los resultados deben interpretarse con cautela y no pueden generalizarse a todas las personas mayores residentes en la Amazonía peruana. Futuras investigaciones deberían considerar diseños longitudinales, muestras probabilísticas y poblaciones más representativas para confirmar los hallazgos observados.

No obstante, el estudio presenta fortalezas importantes. El diagnóstico de sarcopenia fue realizado por personal altamente capacitado, con experiencia clínica y en investigación, utilizando instrumentos validados y equipos que permiten una evaluación precisa. Además, aunque la asociación entre sarcopenia y funcionalidad ha sido previamente estudiada, las comunidades rurales particularmente aquellas localizadas en regiones selváticas han sido escasamente exploradas. Aportar evidencia proveniente de estos contextos contribuye a la toma de decisiones basada en necesidades reales de la población, que pueden diferir según características sociodemográficas, económicas y geográficas.

En conclusión, la sarcopenia se asocia con un incremento en la probabilidad de presentar dependencia funcional para actividades básicas de la vida diaria. Recomendamos que, además de la evaluación rutinaria de la funcionalidad en comunidades rurales, la sarcopenia se incorpore dentro del tamizaje de síndromes geriátricos, dada su relación con diversos desenlaces adversos en salud, incluyendo la dependencia funcional. La identificación temprana permitiría intervenciones oportunas y promover una atención geriátrica centrada en las necesidades individuales, dirigida a preservar la autonomía y mejorar la calidad de vida de las personas mayores.
